# One body, one test, two lives.. patient centered strategy to increase HIV testing in pregnant women and their partners

**DOI:** 10.1186/1742-4690-9-S1-P70

**Published:** 2012-05-25

**Authors:** Larisa Kudryashova-Hernandez

**Affiliations:** 1 Ancillary HIV Services at Neighborhood Health Services Corporation, Plainfield, USA

## Background

NHSC, an urban community-based health center in New Jersey, USA, provides prenatal services, labor/ delivery to 750 uninsured/impoverished/minority women annually. Given that NJ has the third highest number of HIV women in USA and the highest number of HIV children, early HIV detection/ intervention in pregnant women become paramount. NHSC historically struggled with sub-optimal OB HIV testing rates (60%) and needed to make radical program changes to comply with CDC recommendations to ensure HIV testing is offered to 100% pregnant patients.

## Methods

PDSA (Plan-Do-Study-Act) was conducted to test a new HIV testing approach: HIV Counselors are located in OB department; HIV counseling/ Rapid testing is done at OB registration; daily registration schedules are available to HIV Counselors; HIV results become part of OB records upon result availability; educational DVDs are utilized in patient areas to increase awareness/ interest.

## Results

Per PDSA-improved strategy, NHSC sustained 100% compliance with CDC recommendations over the last three years. Rapid HIV testing and Rapid-on-Rapid positive result confirmation allow for smooth/timely transition from HIV testing to care/treatment for newly diagnosed HIV pregnant patients/partners.

## Conclusions

The collected/analyzed data suggests that coordinated, patient-centered approach helps to: identify HIV positive pregnant patients in the first/second trimesters; immediately connect them to prenatal/HIV care to minimize vertical HIV transmission; provide prevention/treatment for partners including prevention for positives.

